# Guidelines for the management of diabetes‐related ketoacidosis (DKA) have been poorly adopted and implemented, resulting in a lack of improvement in outcomes

**DOI:** 10.1111/dme.70010

**Published:** 2025-02-10

**Authors:** Angelica Sharma, Lakshmi Rengarajan, Parth Narendran, Ketan Dhatariya, Punith Kempegowda, DEKODE Working Group, Anu Abraham, Muhammad Ali Karamat, Sanjay Saraf, Nevil C. Philip, Ragavendran Govindaraj Sureshkumar, Manbir Duggal, Saima Kauser Malik, Canay Onder, Kaushal Maru, Pranav Viswanath Iyer, Shivam Choudhary, Ankita Gupta, Anmol Bagga, Carina Synn Cuen Pan, Emily Warmington, Anjitha Anilkumar, Francesca Pang, My Chi Pham, Eka Melson, Meghna Hebbar, Wai Nga Alice Yip, Dineshwaran Rajendran, Shamanth Soghal, Lucy Bomphrey, Dolu Falowo, Senthil Kumar, Catherine Cooper, Wai Nga Alice Yip, Megan Owen, Jason Cheung

**Affiliations:** ^1^ Norfolk and Norwich University Hospitals NHS Foundation Trust Norwich UK; ^2^ Norwich Medical School University of East Anglia Norwich UK; ^3^ Queen Elizabeth Hospital, University Hospitals Birmingham NHS Foundation Trust Birmingham UK; ^4^ Department of Applied Health Sciences University of Birmingham Birmingham UK; ^5^ Institute of Immunology and Immunotherapy University of Birmingham Birmingham UK

**Keywords:** complications, diabetes mellitus, diabetic ketoacidosis, hypoglycaemia, insulin

## Abstract

**Aims:**

The Joint British Diabetes Society‐Inpatient (JBDS‐IP) group recommends reducing fixed rate intravenous insulin infusion (FRIII) from 0.1 to 0.05 units/kg/h when blood glucose falls <14 mmol/L to reduce the risk of complications associated with acute management of diabetes‐related ketoacidosis. However, whether this change results in real‐world improvements is not known.

**Methods:**

We performed a retrospective review of DKA admissions between October 2021 and March 2023 across five hospitals in the United Kingdom. We collated data on demographics, biochemical profiles, management interventions, complications, and outcomes.

**Results:**

We identified 753 DKA admissions. There was a slow uptake of reduced‐rate FRIII, reaching 49.7% over 18 months. In DKA episodes where FRIII rate reduction guidelines were adopted, there was a significant lag (median [IQR] hours) between starting 10% Dextrose and FRIII rate reduction when blood glucose became <14 mmol/L (0.5 (0.1–1.8) vs. 3.2 (0.7–6.5), *p* = 0.00001). There was no significant reduction in hypoglycaemia (16.5% vs. 13.8%, *p* = 0.344) in episodes that adopted FRIII reduction. There were no significant differences in the frequency of hypokalaemia, hyperkalaemia, DKA duration, and length of stay between episodes with FRIII rate reduction versus those without FRIII.

**Conclusions:**

Our study demonstrates suboptimal adoption of guidelines. Therefore, it was perhaps unsurprising that no favourable effect on the rate of complications or outcomes in DKA episodes with reduced‐rate FRIII was demonstrated. In DKA episodes where FRIII rate reduction was adopted, there was a significant delay in adjusting the FRIII when glucose levels were <14 mmol/L. Understanding the barriers and facilitators is vital in creating resources to safely implement guidelines.

**What's new?**

- Previous research indicates that adhering to protocol‐driven DKA management improves outcomes. The Joint British Diabetes Society‐Inpatient (JBDS‐IP) group formulated guidelines for standardised DKA care. However, compliance with JBDS‐IP guidelines in clinical practice and its impact on clinical and patient‐centred outcomes remains insufficiently explored.
- In DKA episodes where fixed‐rate intravenous insulin infusion (FRIII) rate reduction was adopted, there was a significant delay in adjusting the FRIII rate when glucose was <14 mmol/L. We did not find any favourable effect on the rate of complications or outcomes in DKA episodes with reduced‐rate FRIII.
- Understanding factors that influence adherence to guidelines is crucial in creating resources to implement guidelines effectively.

## 1 INTRODUCTION

Diabetes‐related ketoacidosis (DKA) is a potentially life‐threatening yet preventable complication of predominantly type 1 diabetes (T1D).^1^ However, it may also occur in ketosis‐prone type 2 diabetes (T2D) and with the use of novel agents such as sodium‐glucose co‐transporter‐2 (SGLT2) inhibitors.^2^, ^3^ DKA is characterised by a triad of blood glucose concentration >11.0 mmol/L or known diagnosis of diabetes, capillary of blood ketone concentration >3.0 mmol/L or significant ketonuria and bicarbonate concentration of <15.0 mmol/L and/or venous pH <7.3.^4^ DKA may occur in states of absolute or relative insulin deficiency or as a consequence of a rise in counter‐regulatory hormones including cortisol, growth hormone, glucagon and catecholamines. The alteration in hormone levels contributes to elevated blood glucose, lipolysis, ketone body production and electrolyte imbalances.^1^ Mismanagement of DKA may result in increased length of hospital admission and high readmission rates.^4^ Previous studies have demonstrated that protocol‐driven management of DKA is a safe approach—decreasing the duration of DKA and length of hospital admission with no difference in hypoglycaemia and hypokalaemia.^5^

Though the diagnostic criteria for DKA remain unambiguous in the United Kingdom, there remains a variation in DKA management guidelines between hospital sites.^6^, ^7^ To reduce errors and improve the standard of care, the Joint British Diabetes Society‐Inpatient (JBDS‐IP) published national guidelines for DKA management, which have been regularly revised.^4^ Initial surveys have demonstrated a high uptake of guidelines. Management within the emergency department or acute medical unit is initiated in a timely manner, presumably due to a higher nursing staff‐to‐patient ratio and familiarisation with guidelines. However, prompt monitoring and review are often overlooked when patients are transferred to general medical wards.^8^ Rates of continued guideline adherence in clinical practice and, thus, the impact of guideline revisions on outcomes remains less well explored.^9^ A national survey of DKA management demonstrated a high burden of hypoglycaemia and hypokalaemia episodes, with the use of insulin recognised as the primary driver for these biochemical abnormalities.^4^, ^9^ Therefore, based upon expert consensus, the 2021 revision recommends reducing the fixed rate intravenous insulin infusion (FRIII) rate to 0.05 units/kg/h. when blood glucose reduces to less than 14 mmol/L to avoid complications, including hypoglycaemia and hypokalaemia.^4^ However, whether this guideline change has resulted in the expected improvement in DKA care is unknown.

### 1.1 Objectives

1. Determine the rate of uptake of revised guidelines.
2. Study the adherence to guidelines for starting 10% dextrose and FRIII reduction after the first instance of blood glucose <14 mmol/L during a DKA episode.
3. Compare the rates of hypoglycaemia, hypokalaemia, and hyperkalaemia events between DKA episodes that adhered to national guidelines for management vs. those that did not.
4. Analyse baseline characteristics and outcomes of patients who developed hypoglycaemia despite adherence to national DKA recommendations.

## 2 METHODS

### 2.1 Study design and population

We conducted a retrospective analysis of all DKA episodes in individuals aged 16 years or above from October 2021 to March 2023 admitted in five UK hospitals participating in the DEKODE (Digital Evaluation of Ketosis and Other Diabetes Emergencies) initiative. The DEKODE model is a multi‐centre initiative in the UK that analyses DKA management and outcomes.^10^ The model provides regular performance feedback on key factors of DKA care while highlighting areas for improvement compared to the composite median. The model has resulted in a large registry which offers the opportunity to explore the implementation guidelines and their subsequent impact on outcomes.

In the study, two out of the five hospitals are tertiary care centres located in the West Midlands and East of England, each serving populations exceeding 1 million individuals. The remaining three are district general hospitals, each with a catchment area of up to 500,000 people. All hospitals provide services to a diverse, multi‐ethnic population and had an integrated care pathway for DKA management based on JBDS‐IP guidelines.^4^

Participants were categorised into two groups: those who experienced a reduction in the FRIII to 0.05 units/kg/h when their blood glucose levels fell below 14 mmol/L (exposure group), and those who did not receive this adjustment (comparison group).

We further stratified participants by type of diabetes and DKA severity. DKA severity was determined as per JBDS‐IP criteria.^4^

### 2.2 Study parameters and outcomes

We defined DKA as per recommendations by the JBDS‐IP guidelines (capillary or serum glucose >11.0 mmol/L or a history of diabetes, and capillary or serum ketones >3.0 mmol/L or urine ketones ≥ ++ and pH <7.30 or bicarbonate <15.0 mmol).^4^ DKA resolution was defined as pH >7.30 or bicarbonate >18.0 mmol and capillary or serum ketones <0.6 mmol/L for two consecutive hours.

For this study, we identified specific parameters from this database relevant to implementing FRIII reduction guidelines and their impact on outcomes. These included sociodemographic data (age, gender, and ethnicity), duration of DKA, length of hospital admission and rate of FRIII. We also included the date and time of DKA diagnosis, resolution, time of first instance for blood glucose <14 mmol/L during DKA episode, time to start 10% dextrose and time to reduce FRIII to 0.05 units/kg/h. Outcome measures included episodes of hypoglycaemia (defined as <4 mmol/L), hypokalaemia (defined as <3.5 mmol/L), hyperkalaemia (defined as >5.5 mmol/L), and mortality. We evaluated outcomes as they occurred during the DKA episode. DKA duration was calculated as the time difference between DKA diagnosis and resolution and expressed in hours. Length of stay was calculated as the time difference between admission and discharge or death expressed in days.

DKA precipitants were classified into alcohol‐related, COVID‐19, drug‐induced, intercurrent illness, pump failure, new diagnosis of T1D, new diagnosis of T2D, sepsis, sodium‐glucose co‐transporter‐2 inhibitor (SGLT2i) related, suboptimal compliance with treatment, and unknown causes.

We described the ‘adoption’ of guidelines as a reduction in FRIII during the DKA episode and ‘implementation’ when FRIII reduction was adjusted correctly, as per the recommendations.

### 2.3 Statistics

All data were tested for normality of distribution, and results were expressed in median and interquartile range (IQR) for non‐parametric continuous data. Categorical variables were described using proportions and frequencies. The Chi‐square test was used for categorical variables, and the independent t‐test for continuous variables was used to assess statistical significance between groups. Fisher's exact test was used for categorical variables with low cell numbers. The Mann–Whitney U‐test was employed for non‐parametric continuous data, which was not normally distributed. Statistical significance was defined as a *p*‐value < 0.05. Data were analysed using SPSS 29.0.

### 2.4 Ethical considerations

The data was obtained following approval from information governance in respective hospitals (approval numbers: Hospital A, B, D – 12,074; C – DIAB‐22‐23‐A08; E – QI20‐21/LTC/01). All data was pseudonymised at the point of collection.

## 3 RESULTS

### 3.1 Baseline characteristics

We identified 753 DKA episodes from five hospitals (A‐E) during the study period. Of these, 29% (*n* = 218/753) of DKA episodes had appropriate FRIII reduction between October 2021 – March 2023. Baseline characteristics are summarised in Table 1 and precipitating cause(s) of DKA are summarised in Table S1.

**TABLE 1:** Baseline characteristics of all DKA episodes included in the study.

Parameter	(*n* = 753)
**Age, years [Median (IQR)]**	42.0 (28.0–60.0)
**Gender (Male)**	51.5% (*n* = 388)
**Ethnicity (%, *n*)**	
White	70.9% (*n* = 534)
Asian	8.4% (*n* = 63)
Black/Afro‐Caribbean	4.8% (*n* = 36)
Other	15.9% (*n* = 120)
**Length of stay** ^a^ **(days) [Median (IQR)]**	3.5 (2.2–6.8)
**DKA duration** ^b^ **(hours) [Median (IQR)]**	16.5 (10.9–25.3)
**Type of Diabetes (%, *n*)**	
Type 1 Diabetes (T1D)	63.1% (*n* = 475)
Type 2 Diabetes (T2D)	23.9% (*n* = 180)
New diagnosis of T1D	6.6% (*n* = 50)
New diagnosis of T2D	4.6% (*n* = 35)
Type 3c Diabetes	0.9% (*n* = 7)
Latent autoimmune diabetes of adults	0.3% (*n* = 2)
Gestational diabetes mellitus (GDM)	0.6% (*n* = 4)

Abbreviation: IQR, interquartile range.

^a^ Length of stay defined as the time difference between admission and discharge.

^b^ DKA duration defined as the difference in the time from diagnosis (serum glucose ≥11.0 mmol/L, ketones ≥3.0 mmol/L and pH ≤7.3 or bicarbonate ≤15.0 mmol/L) to resolution (serum glucose <11.0 mmol/L, ketones <0.6 mmol/L and pH >7.3 or bicarbonate >15.0 mmol/L), as per JBDS‐IP guidelines.^4^

### 3.2 Rate of uptake of revised guidelines

#### 3.2.1 Rate of uptake of revised FRIII reduction guidelines in hospitals A‐E by quarters

**FIGURE 1:**
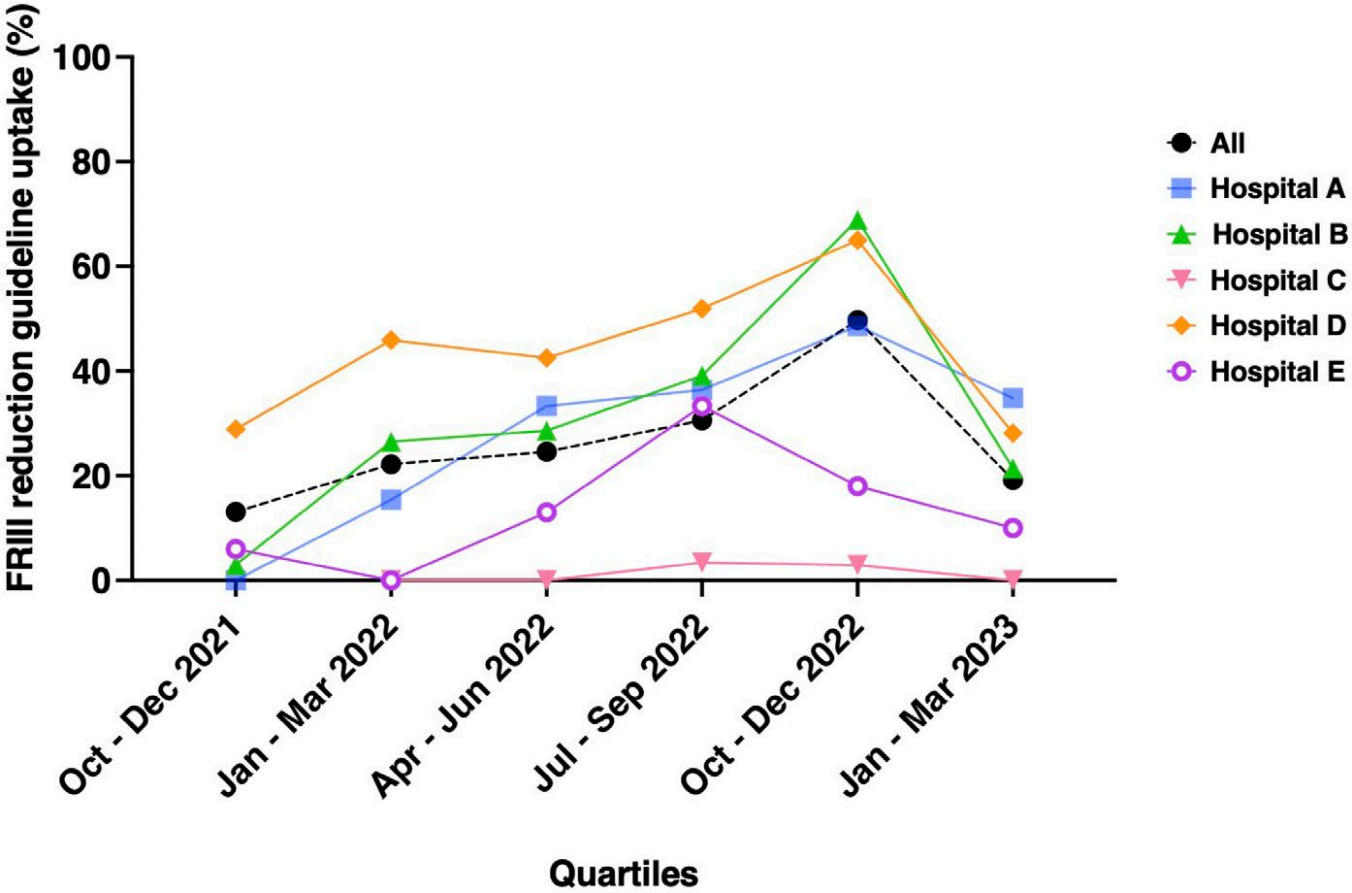
Fixed‐rate intravenous insulin infusion (FRIII) reduction guideline uptake by hospital and quartiles.

During the study period, the uptake of appropriate FRIII dose reduction in DKA management initially improved from 13.5% (*n* = 15/111) to 49.7% (*n* = 92/185) between October 2021 to December 2022 across all hospital sites (Figure 1). Subsequently, there was a reduction to 19.2% (*n* = 20/104) between January – March 2023.

### 3.3 Comparison of the rates of hypoglycaemia, hypokalaemia, and hyperkalaemia events between DKA episodes that adhered to FRIII reduction vs. those that did not

Overall, across all hospital sites, there was no difference in outcomes including hypoglycaemia, hypokalaemia and hyperkalaemia between those individuals who received FRIII reduction vs. those who did not (Table 2).

**TABLE 2:** Frequency of hypoglycaemia,^a^ hypokalaemia^b^ and hyperkalaemia^c^ (%) in diabetic ketoacidosis (DKA) episodes with vs. without fixed rate intravenous insulin infusion rate reduction by 50% initial rate when blood glucose <14 mmol/L during DKA across hospital sites A‐E.

Hospital site	Frequency of hypoglycaemia			Frequency of hypokalaemia			Frequency of hyperkalaemia		
	FRIII rate reduced (*n* = 218)	FRIII rate not reduced (*n* = 535)	*p*‐value	FRIII rate reduced (*n* = 218)	FRIII rate not reduced (*n* = 535)	*p*‐value	FRIII rate reduced (*n* = 218)	FRIII rate not reduced (*n* = 535)	*p*‐value
Overall (*n* = 753) [%, *n*]	17.0% (*n* = 37/218)	13.8% (*n* = 74/535)	0.270	33.5% (*n* = 73/218)	30.7% (164/535)	0.448	29.8% (*n* = 65/218)	29.9% (*n* = 160/535)	0.980
A (*n* = 111) [%, *n*]	9.1% (*n* = 3/33)	12.8% (*n* = 10/78)	0.567	33.3% (*n* = 11/33)	26.9% (*n* = 21/78)	0.496	39.4% (*n* = 13/33)	32.1% (*n* = 25/78)	0.456
B (*n* = 190) [%, *n*]	18.5% (*n* = 12/65)	12.8% (*n* = 16/125)	0.296	41.5% (*n* = 27/65)	40% (*n* = 50/125)	0.838	23.1% (*n* = 15/65)	32% (*n* = 40/125)	0.198
C (*n* = 168) [%, *n*]	50% (*n* = 1/2)	23.9% (*n* = 32/166)	0.199	50% (*n* = 1/2)	20.5% (*n* = 34/166)	0.306	50% (*n* = 1/2)	22.3% (*n* = 37/166)	0.352
D (*n* = 238) [%, *n*]	18.2% (*n* = 20/110)	7.8% (*n* = 10/128)	0.016	28.2% (*n* = 31/110)	31.3% (*n* = 40/128)	0.606	30% (*n* = 33/110)	37.5% (*n* = 48/128)	0.223
E (*n* = 46) [%, *n*]	12.5% (*n* = 1/8)	15.8% (*n* = 6/38)	0.814	37.5% (*n* = 3/8)	50% (*n* = 19/38)	0.520	37.5% (*n* = 3/8)	26.3% (*n* = 10/38)	0.523

^a^ Hypoglycaemia – defined as glucose <4 mmol/L.

^b^ Hypokalaemia – defined as potassium <3.5 mmol/L.

^c^ Hyperkalaemia – defined as potassium >5.5 mmol/L.

#### 3.3.1 Rate of hypoglycaemia in hospital(s) A‐E

Overall, there was no significant difference in the rate of hypoglycaemic episodes between DKA episodes where FRIII was reduced when blood glucose <14 mmol/L (16.5% (*n* = 36/218)) vs. where it was not (13.8% (*n* = 74/535)), *p* = 0.344. This was noted across all hospital sites except Hospital D, where there was a higher rate of hypoglycaemia in DKA episodes where FRIII was reduced (Table 2).

The median (IQR) time from DKA diagnosis to the first hypoglycaemia episode was 11.7 (5.9–19.3) h. Of the 26 episodes of hypoglycaemia that happened in those where FRIII was reduced, 30.8% (*n* = 8) had hypoglycaemia before FRIII rate reduction, 30.8% (*n* = 8) had hypoglycaemia both before and following FRIII reduction, and 38.5% (*n* = 10) had hypoglycaemia event following FRIII reduction. Among the 18 individuals with hypoglycaemia post‐FRIII rate reduction, the minimum time to hypoglycaemia event was 2.9 h, with a median (IQR) of 8.4 (5.6–11.6) h.

#### 3.3.2 Rate of hypokalaemia in hospital(s) A‐E

There was no significant difference in episodes of hypokalaemia between DKA episodes where FRIII reduction was appropriately implemented (33.5% (*n* = 73/218)) vs. those where it was not (30.7% (*n* = 164/535)), *p* = 0.448. This was noted across all hospital sites (Table 2).

#### 3.3.3 Rate of hyperkalaemia in hospital(s) A‐E

There was no significant difference in episodes of hyperkalaemia between DKA episodes where FRIII reduction was appropriately implemented (29.4% (*n* = 64/218)) vs. those where it was not (29.9% (*n* = 160/535)), *p* = 0.881. This was unanimous across all hospital sites (Table 2).

#### 3.3.4 Mortality in hospital(s) A‐E

Overall, across all hospital sites, there was no difference in mortality (Table 3).

**TABLE 3:** Difference in mortality between those who received FRIII rate reduction vs. those who did not.

Hospital site	Mortality		
	FRIII rate reduced (*n* = 218)	FRIII rate not reduced (*n* = 535)	*p*‐value
All hospital sites (*n* = 753) [%, *n*]	3.7% (*n* = 8/218)	4.1% (*n* = 22/535)	0.778
A (*n* = 111) [%, *n*]	0.0% (*n* = 0/33)	3.8% (*n* = 3/78)	0.253
B (*n* = 190) [%, *n*]	1.5% (*n* = 1/65)	4.8% (*n* = 6/125)	0.258
C (*n* = 168) [%, *n*]	50.0% (*n* = 1/2)	4.2% (*n* = 7/166)	0.093
D (*n* = 238) [%, *n*]	5.5% (*n* = 6/110)	4.7% (*n* = 6/128)	0.787
E (*n* = 46) [%, *n*]	0.0% (*n* = 0/8)	0.0% (*n* = 0/38)	1.000

### 3.4 Comparison of length of hospital admission and DKA duration between DKA episodes that adhered to FRIII reduction vs. those that did not

Overall, across all hospital sites, there was no difference in length of stay or duration of DKA between individuals who received FRIII reduction and those who did not (Table 4).

**TABLE 4:** Total duration of diabetic ketoacidosis (DKA) and length of stay in episodes with vs. without fixed rate intravenous insulin infusion rate reduction by 50% initial rate when blood glucose <14 mmol/L during DKA across hospital sites A–E.

Hospital site	DKA Duration (hours)			Length of stay (days)		
	FRIII rate reduced (*n* = 218)	FRIII rate not reduced (*n* = 535)	*p*‐value	FRIII rate reduced	FRIII rate not reduced	*p*‐value
All hospital sites (*n* = 753) (Median [IQR])	16.9 (11.8–26.8)	17.0 (11.8–26.9)	0.667	3.4 (2.2–6.3)	3.4 (2.2–6.3)	0.749
A (*n* = 111) (Median [IQR])	14.7 (12.9–19.0)	12.4 (8.4–17.0)	0.004	3.4 (2.2–5.0)	2.8 (1.8–5.2)	0.265
B (*n* = 190) (Median [IQR])	19.9 (14.5–30.5)	18.6 (12.9–27.5)	0.522	3.5 (2.7–5.1)	4 (2.5–7)	0.554
C (*n* = 168) (Median [IQR])	19.2, 41.7*	22.8 (16.3–39.9)	0.879	6.7, 7.4*	3.3 (2.0–7.2)	0.378
D (*n* = 238) (Median [IQR])	15.8 (10.7–26.6)	13.5 (9.7–20.8)	0.078	3.4 (2.2–6.2)	3.8 (2.3–6.2)	0.819
E (*n* = 46) (Median [IQR])	18.0 (16.6–24.0)	19.6 (11.8–25.0)	0.573	2.9 (2.0–3.2)	3.3 (2.1–7.6)	0.303

*Note*: All results are expressed as median (interquartile range [IQR]), except hospital C, which only had two DKA episodes where the fixed rate was reduced; hence, the results for this are presented as separate data items and highlighted with an asterisk (*).

#### 3.4.1 DKA duration

There was no difference in the overall median (IQR) duration of DKA (hours) between DKA episodes where FRIII reduction was appropriately implemented (17 (12–25)) vs. not appropriately implemented (17 (11–27)), *p* = 0.750. At hospital site A, there was a longer median (IQR) DKA duration (hours) (14.7 (12.9–19)) in those who had a reduction in FRIII vs. those who did not (12.4 (8.4–17)), *p* = 0.004 (Table 4).

#### 3.4.2 Length of hospital admission

There was no notable difference in length of hospital admission (days) between DKA episodes where FRIII reduction was reduced (3.4 (2.4–5.6)) vs. not reduced (3.4 (2.1–6.8)), *p* = 0.753. This was noted across all hospital sites (Table 4).

### 3.5 Adherence to guidelines for starting 10% dextrose and FRIII reduction after the first instance of blood glucose <14 mmol/L during DKA

Of the 218 DKA events that adopted FRIII reduction, we retrieved complete data for implementing FRIII reduction for 180 DKA events. There was no significant difference in baseline characteristics between those that developed hypoglycaemia following FRIII reduction vs. those that did not (Table S2). There was a trend for longer DKA episodes [hours] (23.7 (13.6–31.8) vs. 16.2 (10.8–24.4), *p* = 0.060) and higher total units of FRIII administered during DKA episodes (152.7 (81.3–254.3) vs. 115.8 (64.7–192.8), *p* = 0.085) in those with hypoglycaemic events vs. those without (Table 5).

**TABLE 5:** Biochemical profiles and outcomes of diabetic ketoacidosis (DKA) episodes with fixed rate intravenous insulin infusion (FRIII) rate reduction by 50% initial rate when blood glucose <14 mmol/L during DKA.

Parameter	FRIII rate reduction (*n* = 180)	No hypoglycaemia (*n* = 154)	Hypoglycaemia (*n* = 26)	*p*‐value
pH	7.2 (7.1–7.3)	7.2 (7.1–7.3)	7.2 (7.1–7.3)	0.826
Bicarbonate (mmol/L) [Median (IQR)]	11.4 (7.9–15)	11.5 (8.3–15.0)	10.4 (7.5–15.1)	0.373
Glucose (mmol/L) [Median (IQR)]	24.4 (19.6–31.8)	24.4 (20.0–31.8)	21.4 (17.6–31.5)	0.503
Ketones (mmol/L) [Median (IQR)]	5.9 (5.0–7.0)	5.9 (5.0–7.0)	6.2 (5.0–7.4)	0.617
Lactate (mmol/L) [Median (IQR)]	2.5 (1.8–3.8)	2.6 (1.8–3.8)	2 (1.7–3.5)	0.280
Hypokalaemia episodes [K < 3.5] (%, *n*)	4.4% (*n* = 8)	3.9% (*n* = 6)	7.7% (*n* = 2)	0.384
Hyperkalaemia episodes [K > 5.5] (%, *n*)	19.4% (*n* = 35)	21.4% (*n* = 33)	7.7% (*n* = 2)	0.102
Urea (mmol/L)	7.2 (5.1–11.8)	7.2 (5.1–11.8)	6.6 (5.1–12.9)	0.952
DKA duration (hours) [Median (IQR)]	16.7 (11.3–25.3)	16.2 (10.8–24.4)	23.7 (13.6–31.8)	0.060
Length of hospital stay (days) [Median (IQR)]	3.9 (2.6–7.1)	4 (2.6–7.0)	3.3 (2.6–6.0)	0.826
Total FRIII administered during DKA (units) [Median (IQR)]	117.9 (64.9–200.0)	115.8 (64.7–192.8)	152.7 (81.3–254.3)	0.085

Abbreviation: IQR, interquartile range.

When blood glucose levels drop below 14 mmol/L during the DKA episode, guidelines recommend commencing 10% Dextrose alongside FRIII reduction. However, there was a significant lag between starting 10% Dextrose and FRIII reduction during DKA episodes (median [IQR] hours − all episodes: 0.5 (0.1–1.8) vs. 3.2 (0.7–6.5), *p* = 0.00001; DKA episodes with hypoglycaemia: 0.6 (0.2–3.1) vs. 3.5 (1.2–8.9), *p* = 0.003; DKA episodes without hypoglycaemia: 0.5 (0.1–1.6) vs. 3.2 (0.7–6.2), *p* = 0.00001) (Table 6).

**TABLE 6:** Time intervals during diabetic ketoacidosis (DKA) episode in FRIII rate reduction group.

Parameter	FRIII reduction (*n* = 180)	No hypoglycaemia (*n* = 154)	Hypoglycaemia (*n* = 26)	*p*‐value
Time of DKA onset to glucose <14 mmol/L, hours (Median [IQR])	3.9 (2.4–6.5)	4.0 (2.6–7.1)	2.8 (1.6–4.5)	0.004
Time from glucose <14%–10% Dextrose administration, hours (Median [IQR])	0.5 (0.1–1.8)	0.5 (0.1–1.6)	0.6 (0.2–3.1)	0.427
Time from glucose <14 mmol/L to FRIII reduction, hours (Median [IQR])	3.2 (0.7–6.5)	3.2 (0.7–6.2)	3.5 (1.2–8.9)	0.243
Time from DKA diagnosis to hypoglycaemia, hours (Median [IQR])	–	–	11.7 (5.9–19.3)	–

Abbreviation: IQR, interquartile range.

### 3.6 Outcomes stratified by DKA severity and type of diabetes

Within the total population, 47.4% (*n* = 357/753) experienced a severe DKA episode. However, there were no differences in outcomes between individuals who received FRIII reduced only; received 10% Dextrose only; received FRIII reduction and received 10% Dextrose nor those who did not receive FRIII reduction and did not receive 10% Dextrose (Table S3). This was also noted for individuals who developed simple DKA (Table S3).

We further stratified the population by type of diabetes: 69.7% (*n* = 525/753) of individuals had T1D and 28.6% (*n* = 215) had T2D. We excluded individuals with other types of diabetes for this analysis (*n* = 13/753). Amongst individuals with T1D and T2D, there were no differences in outcomes between those who had FRIII reduced only; received 10% Dextrose only; received FRIII reduced and received 10% Dextrose nor those who did not have FRIII reduced and did not receive 10% Dextrose (Table S4).

The significant difference in mortality across all subgroups and hypoglycaemia in T2D is likely to be due to small sample sizes.

## 4 DISCUSSION

Our findings highlight a slow uptake of guidelines, with less than half of individuals receiving care as per guideline change even 18 months after the guideline revision. There were no appreciable differences in biochemical outcomes of hypoglycaemia, hypokalaemia and hyperkalaemia following the guideline introduction of FRIII reduction. In addition, a reduction in FRIII did not affect the overall DKA duration, length of hospital admission nor mortality. This was also noted upon stratification by DKA severity and type of diabetes. Administration of 10% Dextrose as per guidelines when glucose levels were below 14 mmol/L took a median time of 30 minutes. However, FRIII reduction was delayed by over 3 h within our cohort. Therefore, none of the DKA episodes in our study had appropriate implementation of DKA guidelines.

Theoretically, a reduction in FRIII may result in a longer duration of DKA and consequently length of hospital admission due to slower ketone clearance blood glucose normalisation. This rationale aligns with our observations that individuals receiving FRIII rate reduction tend to exhibit a pattern of prolonged DKA duration and extended hospital stay as compared to their counterparts who do not receive FRIII reduction. However, it is important to note that the purpose of reducing FRIII during a DKA episode is to minimise the life‐threatening risk of hypoglycaemia and hypokalaemia. Phillips and Sinha reported a significant reduction in the frequency of hypoglycaemia during DKA after reducing the rate of FRIII to 0.05 units/kg/h. When blood glucose falls below 12 mmol/L.^11^ Their findings support the effectiveness of this approach and were further advocated by the revised JBDS‐IP guidelines in 2021. However, our study found a sluggish adoption of guidelines in all hospitals, and when adopted, management was not initiated promptly. This may explain the lack of appreciable differences in the frequency of hypoglycaemia, hypokalaemia and hyperkalaemia following FRIII reduction. Mohamed et al. reported that the effective incorporation of a standardised protocol for managing DKA, with 87% managed as per the guideline, substantially enhanced adherence to best practices in care.^12^ This underscores the necessity for a well‐defined implementation strategy and continuous monitoring or auditing principles to ensure the delivery of appropriate patient care.

The effects of clinical care pathways are well established.^13^, ^14^ Waller et al. found that clinical care pathways for DKA significantly improved key areas in managing DKA, although there remained room for further improvements.^15^ Introducing a DKA protocol resulted in a 23% reduction in intensive care unit stay, earlier resolution of ketosis with no increase in the rate of hypoglycaemia and a 30% reduction in hospital stay.^16^ A study of adults admitted with DKA to the Medicine and Critical Care Services in a US teaching hospital found that introducing a DKA critical pathway reduced length of stay (5.2 vs. 2.4 days, respectively) and hospital costs even in individuals managed without specialist endocrinology input.^17^

In a UK‐based retrospective study, initial adherence to DKA guidelines was reported to be over 70%.^5^ However, a review of electrolytes was only performed in 38% after 2–4 h of management, and 35%–60% had correct fluids prescribed.^5^ Possible reasons included the absence of well‐defined parameters for escalating management to higher level care, insufficient clinician knowledge of protocols and inefficient patient handover during ward transfer. Several factors may impact the implementation of guidelines including patient: healthcare professional staff ratios, lack of educational material, access to electronic health records and availability of specialised staff including pharmacists and diabetes specialist nurses. The DEKODE model aims to address this issue by promoting performance‐based quarterly feedback on these areas to enable the application of quality improvement techniques in real time.^18^ The continuous audit process with individualised feedback ensures that each participating hospital site can target educational material and initiatives to areas and teams which require the most support.

The strengths of our study include its multi‐centre design and inclusion of standardised biochemical and outcome data alongside adherence rates. Though no formal sample size was calculated, we included all DKA episodes during the study period to conduct the preliminary data analysis for this exploratory study to inform future research directions. The lack of significant improvement in complication rate may be attributed to the small sample size within our study. However, to the best of our knowledge, this is the largest dataset analysing adherence to and the impact of clinical guideline change in DKA management. Our study is limited by its retrospective nature and lack of data on co‐morbidities. A thorough search of case notes and online clinical systems with the assistance of the local hospital informatics team minimised missing data.

This study utilised data from the DEKODE registry, a comprehensive database designed to support the exploration of diverse research questions in diabetes‐related emergencies. While this registry has been used in previous analyses to address distinct research objectives (Ref: DME‐2024‐xxxx), the present study focuses on adherence to fixed‐rate intravenous insulin reduction guidelines. This approach aligns with established epidemiological and registry‐based research practices, where large databases are leveraged for multiple investigations to maximise their utility and generate novel insights. To ensure methodological rigour and minimise overlap, our study adhered to pre‐defined objectives and robust statistical methodologies tailored to the specific hypotheses under investigation.

Further qualitative research is urgently needed to ascertain the reasons behind the low adherence rate towards guidelines, whether complication rates may be lower with higher guideline uptake and the use of guidelines in individuals with recurrent DKA admissions in order to optimise patient outcomes.

## 5 CONCLUSION

Fewer DKA episodes in our study received a reduction in FRIII as per JBDS‐IP guidelines, even over 18 months following the update in respective hospitals. In DKA episodes where FRIII rate reduction was implemented, there was a significant delay in adjusting the FRIII when glucose levels were <14 mmol/L. We did not find any favourable effect on the rate of complications nor outcomes in DKA episodes with reduced‐rate FRIII, possibly owing to a suboptimal implementation of the guidelines. Further work is required to understand the clinical and human factors involved in clinical guideline adherence and the lack of clinical gain from this guideline change.

## AUTHOR CONTRIBUTIONS

AS and LR were equally involved in data collection, analysis, and manuscript writing and share joint first authorship. PN and KD provided critical input on the study design, analysis, and manuscript writing. PK conceptualised and supervised the study's design, data collection, analysis, and manuscript writing. The members of the DEKODE working group were involved in data collection and implementation of guidelines. All of them qualify for shared authorship of this paper.

## FUNDING INFORMATION

This research received no specific grant from any funding agency in the public, commercial or not‐for‐profit sectors. PK receives support from the National Institute for Health and Care Research (NIHR) through his Advanced Clinician Scientist Fellowship (NIHR303671). Additionally, he is supported by the Midlands Patient Safety Research Collaboration (PSRC), and the NIHR‐supported Race, Equity, and Diversity in Careers Incubator. The views expressed in this study are those of the authors and do not necessarily reflect the official positions of the NIHR or the Department of Health and Social Care.

## CONFLICT OF INTEREST STATEMENT

The authors declare no conflicts of interest.

## Supporting information


Table S1.

Table S2.

Table S3.

Table S4.
