# Supplementary material for: Guidelines for the management of diabetes‐related ketoacidosis (DKA) have been poorly adopted and implemented, resulting in a lack of improvement in outcomes
Source: Diabet Med. 2025 Feb 10;42(6):e70010. doi: 10.1111/dme.70010 (PMC12080986; doi:10.1111/dme.70010)
Supplement: Supplementary file 1 — Table S1. [file DME-42-e70010-s001.docx]

*Supplementary Table 1:* Precipitating cause of DKA episodes included in the study.

| **Precipitating cause** | **(n=753)** |
| --- | --- |
| Alcohol-related | 3.1% (n=23) |
| COVID | 3.5% (n=26) |
| Drug-induced | 0.9% (n=7) |
| Intercurrent illness | 33.7% (n=254) |
| Pump failure | 1.2% (n=8) |
| New diagnosis of T1D | 6.6% (n=50) |
| New diagnosis of T2D | 4.6% (n=35) |
| Sepsis | 4.9% (n=37) |
| Sodium-glucose co-transporter-2 inhibitor (SGLT2i) related | 2.4% (n=18) |
| Suboptimal compliance to treatment | 31.3% (n=236) |
| Unknown | 7.8% (n=59) |
