# Supplementary material for: Guidelines for the management of diabetes‐related ketoacidosis (DKA) have been poorly adopted and implemented, resulting in a lack of improvement in outcomes
Source: Diabet Med. 2025 Feb 10;42(6):e70010. doi: 10.1111/dme.70010 (PMC12080986; doi:10.1111/dme.70010)
Supplement: Supplementary file 2 — Table S2. [file DME-42-e70010-s003.docx]

*Supplementary Table 2:* Baseline characteristics of diabetic ketoacidosis (DKA) episodes with fixed rate intravenous insulin infusion rate reduction by 50% initial rate when blood glucose <14 mmol/L during DKA.

| **Parameter** | **FRIII rate reduction (n=180)** | **No hypoglycaemia (n=154)** | **Hypoglycaemia (n=26)** | **p-value** |
| --- | --- | --- | --- | --- |
| **Age (years)** | 37.0 (28.0 – 59.0) | 37.0 (27.0 – 58.0) | 37.0 (32.0 – 64.0) | 0.423 |
| **Gender (Male)** | 56% (n=101) | 57.8% (n=89) | 46.2% (n=12) | 0.269 |
| **Ethnicity [%, n]** |  |  |  | 0.732 |
| *White* | 68.3% (n=123) | 66.9% (n=103) | 76.9% (n=20) |  |
| *Asian* | 10.6% (n=19) | 11% (n=17) | 7.7% (n=2) |  |
| *Black* | 7.8% (n=14) | 7.8% (n=12) | 7.7% (n=2) |  |
| *Others* | 13.3% (n=24) | 14.3% (n=22) | 7.7% (n=2) |  |
| **Body Mass Index (kg/m^2^)** | 23.9 (20.8 – 29.1) | 24.2 (20.9 – 29.5) | 23.5 (19.8 – 27.7) | 0.542 |
