# Supplementary material for: Guidelines for the management of diabetes‐related ketoacidosis (DKA) have been poorly adopted and implemented, resulting in a lack of improvement in outcomes
Source: Diabet Med. 2025 Feb 10;42(6):e70010. doi: 10.1111/dme.70010 (PMC12080986; doi:10.1111/dme.70010)
Supplement: Supplementary file 3 — Table S3. [file DME-42-e70010-s002.docx]

*Supplementary Table 3:* Outcomes in individuals who adopted FRIII reduction guidelines in simple DKA and severe DKA vs those who did not

| **Simple DKA (n=396)** | | | | | |
| --- | --- | --- | --- | --- | --- |
| **Outcome** | **FRIII reduced only**  **(n=5)** | **Dextrose started only**  **(n=266)** | **FRIII reduced and Dextrose started (n=109)** | **FRIII not reduced and Dextrose not started (n=16)** | **p-value** |
| **Hypoglycaemia** | 20.0% (n=1) | 15.0% (n=40) | 18.3% (n=20) | 6.3% (n=1) | 0.611 |
| **Hypokalaemia** | 40.0% (n=2) | 24.8% (n=66) | 35.8% (n=39) | 50.0% (n=8) | 0.038 |
| **Length of stay (days) [Median (IQR)]** | 3.0 (2.9 – 3.6) | 3.3 (2.0 – 6.9) | 3.0 (2.23 – 5.2) | 4.2 (2.8 – 8.6) | 0.694 |
| **Duration of DKA (hours) [Median (IQR)]** | 15.1 (10.8 – 16.0) | 17.5 (10.9 – 27.0) | 15.8 (10.4 – 28.7) | 13.5 (10.4 – 19.7) | 0.379 |
| **Mortality** | 40% (n=2) | 4.1% (n=11) | 0.9% (n=1) | 6.3% (n=1) | 0.001 |
| **Severe DKA (n=357)** | | | | | |
| **Outcome** | **FRIII reduced only (n=6)** | **Dextrose started only (n=232)** | **FRIII reduced and Dextrose started (n=98)** | **FRIII not reduced and Dextrose not started (n=21)** | **p-value** |
| **Hypoglycaemia** | 50.0% (n=3) | 12.5% (n=29) | 13.3% (n=13) | 14.3% (n=3) | 0.069 |
| **Hypokalaemia** | 66.7% (n=4) | 36.2% (n=84) | 28.6% (n=28) | 28.6% (n=6) | 0.179 |
| **Length of stay (days) [Median (IQR)]** | 8.6 (3.6 – 12.5) | 3.3 (2.2 – 6.3) | 4.0 (2.6 – 6.3) | 4.5 (3.3 – 6.4) | 0.244 |
| **Duration of DKA (hours) [Median (IQR)]** | 24.2 (17.6 – 25.5) | 17.9 (12.4 – 28.2) | 17.3 (13.3 – 24.6) | 13.6 (11.8 – 23.1) | 0.453 |
| **Mortality** | 16.7% (n=1) | 3.9% (n=9) | 4.1% (n=4) | 4.8% (n=1) | 0.494 |
