# Supplementary material for: Guidelines for the management of diabetes‐related ketoacidosis (DKA) have been poorly adopted and implemented, resulting in a lack of improvement in outcomes
Source: Diabet Med. 2025 Feb 10;42(6):e70010. doi: 10.1111/dme.70010 (PMC12080986; doi:10.1111/dme.70010)
Supplement: Supplementary file 4 — Table S4. [file DME-42-e70010-s004.docx]

*Supplementary Table 4:* Outcomes in individuals with T1D and T2D who adopted FRIII reduction guidelines vs those who did not

| **Type 1 diabetes (T1D) (n=525)** | | | | | |
| --- | --- | --- | --- | --- | --- |
| **Outcome** | **FRIII reduced only (n=10)** | **Dextrose started only (n=340)** | **FRIII reduced and Dextrose started (n=152)** | **FRIII not reduced and Dextrose not started (n=23)** | **p-value** |
| **Frequency of hypoglycaemia (%, n)** | 40.0% (n=4) | 15.3% (n=52) | 18.4% (n=28) | 8.7% (n=2) | 0.121 |
| **Frequency of hypokalaemia (%, n)** | 50.0% (n=5) | 29.7% (n=101) | 31.6% (n=48) | 30.4% (n=7) | 0.580 |
| **Length of stay (days) [Median (IQR)]** | 3.9 (2.2 – 8.8) | 3.0 (1.9 – 5.2) | 3.0 (2.1 – 4.9) | 4.0 (3.2 – 4.9) | 0.491 |
| **Duration of DKA (hours) [Median (IQR)]** | 16.5 (12.0 – 24.8) | 16.7 (10.8 – 24.8) | 15.7 (11.6 – 25.4) | 13.1 (10.3 – 23.2) | 0.641 |
| **Mortality (%, n)** | 20.0% (n=2) | 2.4% (n=8) | 2.6% (n=4) | 4.3% (n=1) | 0.011 |
| **Type 2 Diabetes (T2D) (n=215)** | | | | | |
| **Outcome** | **FRIII reduced only (n=1)** | **Dextrose started only (n=147)** | **FRIII reduced and Dextrose started (n=55)** | **FRIII not reduced and Dextrose not started (n=12)** | **p-value** |
| **Frequency of hypoglycaemia (%, n)** | 100.0% (n=1) | 10.2% (n=15) | 7.3% (n=4) | 8.3% (n=1) | 0.021 |
| **Frequency of hypokalaemia (%, n)** | 100.0% (n=1) | 27.2% (n=40) | 29.0% (n=16) | 50.0% (n=6) | 0.157 |
| **Length of stay (days) [Median (IQR)]** | 2.9 | 5.0 (3.0 – 8.9) | 5.0 (3.6 – 8.1) | 9.8 (5.0 – 12.8) | 0.279 |
| **Duration of DKA (hours) [Median (IQR)]** | 18.0 | 19.0 (13.6 – 32.0) | 19.1 (14.5 – 25.2) | 14.1 (12.4 – 19.4) | 0.460 |
| **Mortality (%, n)** | 100.0% (n=1) | 8.2% (n=12) | 1.8% (n=1) | 8.3% (n=1) | 0.001 |
